# Discovery of calcium-binding peptides derived from defatted lemon basil seeds with enhanced calcium uptake in human intestinal epithelial cells, Caco-2

**DOI:** 10.1038/s41598-022-08380-0

**Published:** 2022-03-18

**Authors:** Norhameemee Kheeree, Kittisak Kuptawach, Songchan Puthong, Papassara Sangtanoo, Piroonporn Srimongkol, Patamalai Boonserm, Onrapak Reamtong, Kiattawee Choowongkomon, Aphichart Karnchanatat

**Affiliations:** 1 Program in Biotechnology, Faculty of Science, Chulalongkorn University, 254 Phayathai Road, Pathumwan, Bangkok, 10330 Thailand; 2 Research Unit in Bioconversion/Bioseparation for Value-Added Chemical Production, Institute of Biotechnology and Genetic Engineering, Chulalongkorn University, 254 Phayathai Road, Pathumwan, Bangkok, 10330 Thailand; 3 Department of Microbiology, Faculty of Science, King Mongkut’s University of Technology Thonburi, 126 Pracha Uthit Road, Tungkru, Bangkok 10140 Thailand; 4 Department of Molecular Tropical Medicine and Genetics, Faculty of Tropical Medicine, Mahidol University, 420/6 Ratchawithi Road, Ratchathewi, Bangkok, 10400 Thailand; 5 Department of Biochemistry, Faculty of Science, Kasetsart University, 50 Ngamwongwan Road, Chatuchak, Bangkok, 10900 Thailand

**Keywords:** Biochemistry, Biotechnology, Plant sciences, Chemistry

## Abstract

It is anticipated that calcium-chelating peptides may serve to enhance the absorption of calcium. This research examined defatted lemon basil seeds (DLBS) which had been treated with Alcalase under optimized parameters for the degree of hydrolysis for proteolysis, discovering that the activity for calcium-binding in a competitive condition with phosphate ion was 60.39 ± 1.545%. The purification of the hydrolysates was performed via ultrafiltration along with reversed-phase high performance liquid chromatography (RP-HPLC). Determination of the purified peptide amino acid sequence was confirmed for both peptides and reported as Ala-Phe-Asn-Arg-Ala-Lys-Ser-Lys-Ala-Leu-Asn-Glu-Asn (AFNRAKSKALNEN; Basil-1), and Tyr-Asp-Ser-Ser-Gly-Gly-Pro-Thr-Pro-Trp-Leu-Ser-Pro-Tyr (YDSSGGPTPWLSPY; Basil-2). The respective activities for calcium-binding were 38.62 ± 1.33%, and 42.19 ± 2.27%. Fluorescence spectroscopy, and fourier transform infrared spectroscopy were employed in order to assess the chelating mechanism between calcium and the peptides. It was found that the calcium ions took place through the activity of the amino nitrogen atoms and the oxygen atoms on the carboxyl group. Moreover, both of these peptides served to improve calcium transport and absorption in Caco-2 cell monolayers, depending on the concentration involved. It was revealed that the peptide-calcium complexes offered an increased calcium absorption percentage when compared to free calcium at similar concentrations. It might be concluded that the peptide within the peptide-calcium complex can promote calcium absorption through both active and passive transport pathways by increasing calcium concentration and promoting cell membrane interaction. Accordingly, DLBS protein can be considered a strong potential source of protein which can be used to produce calcium-binding peptides and might therefore play a role in the production of nutraceutical foods as a bioactive ingredient.

## Introduction

Calcium plays a critical nutritional role in the human body to support a number of biological functions, including mitosis, nerve conduction, blood coagulation, muscle contraction, and skeletal structural support. If the body receives inadequate levels of calcium, the outcome can be reduced bone density, osteoporosis, or rickets^1–4^. It has been suggested that organic calcium would be better than inorganic calcium since there are two key problems with the latter: at low concentrations the bioavailability is poor, and at high concentrations it presents the issue of biological toxicity. Calcium ions and organic ligands are able to produce complexes^5,6^. In general, molecules which attract biominerals must possess a structure which facilitates calcium binding. Polycarboxylic acid or polyphosphoric acid would be suitable examples of this^7^. Phosphoseryl and carboxylic groups are commonly found in casein phosphopeptides (CPPs) as well as in citrate, and are capable of chelating calcium. It is still possible, however, for peptides or proteins which are not associated with phosphate groups to play a role in the absorption of calcium because they are able to bind the calcium through the amino group or via the carboxyl groups found in amino acid residues^8,9^. To date, the principal method of increasing calcium accumulation has been to intake calcium ions from the diet, but it has not correlated with the calcium absorption because of the calcium precipitated during the digestion process. Therefore, it is necessary to discover an alternative dietary supplement for enhancing both calcium absorption and its bioavailability^10^.

The binding potential of bioactive peptides with calcium ions leads to the creation of stable complexes, which act as exceptional supplements. In contrast to alternative calcium supplements, the absorptivity of peptide chelators is superior, while energy consumption is low^11^. Earlier work revealed that when small peptides from dairy products and some protein hydrolysates from food sources are bound to calcium ions, it is possible for the small intestine to absorb them whole^8–10^. While certain organic calcium supplements can be toxic, those peptide chelators which are obtained from food proteins are typically safe as well as known to provide nutritional benefits. Furthermore, there is a tendency for peptide chelators to produce unwanted color reactions while also promoting fat oxidation, and for this reason, they have not found widespread use and are rather unpopular^12^. In general, calcium-binding peptides can easily be obtained via the human diet when health properties are taken into consideration. CPPs and phosphorylated peptides which can be obtained from milk are widely understood to enhance the absorption of calcium and play a major role in mineral binding. In Caco-2 cells, CPPs can improve the uptake of calcium through the activity of the transient receptor potential cation of the vanilloid sub-family V member 6, TRPV6 channel, which is also known as calcium transporter-1, or CaT1^13,14^. Calcium absorption is also supported by fish and meat as part of the diet, because when the muscle tissue is digested, this releases calcium-binding peptides. For some people, the cost of animal protein, and the lactose intolerance are the problems with calcium intake^15^. So, the protein from plant sources is interesting. Many studies found that the peptide from mung bean^16^, wheat germ hydrolysates^17^, and soybean peptide^18^ could interact with calcium ions and lead to an improvement in calcium intake. It is therefore important for these people to apply the plant-base source.

In Thailand, lemon basil seeds (*Ocimum citriodorum*) are a common dessert, popular for its health properties. The seeds are immersed in water to swell the outer pericarp until a gelatinous layer is formed, known as mucilage. These seeds were eaten as a popular in Southeast Asian desserts^19–21^. The mucilage from basil has a high fiber content, which offers benefits to the excretory system. In earlier research, oil was removed from the lemon basil seeds using the supercritical carbon dioxide approach, resulting in 90% unsaturated fatty acid, while one by-product is the protein, making up a high percentage of 57.16%^22^. These by-products are protein-rich as a consequence of the high quality of the protein content in the DLBS oil process. Kheeree et al.^23^ make the point that DLBS protein is also very rich in all of the essential amino acids, which are critical since the amino acid composition of a peptide or hydrolysate governs the biological activities which take place, such as the capacity for calcium binding. One additional property of DLBS protein is its high content of glutamic acid and aspartic acid, for which there is strong evidence of enhanced binding with metal ions. Few studies to date, however, have specifically focused on calcium-binding peptides which can be isolated from DLBS, so this research study has the aim of isolating and characterizing particular calcium-binding peptides obtained from DLBS protein, as well as developing a deeper understanding of the potential chelating mechanism. It is possible to use the human adenocarcinoma intestinal cells, Caco-2 cells as a model of the intestinal epithelial barrier to assess the uptake of calcium. This research therefore proposes the use of DLBS protein as a source of calcium-binding peptides which might play a useful nutraceutical role to support the health of human bones in the future.

## Results and discussion

### Effect of alcalase on calcium-binding activity

Enzymatic protein hydrolysis is based on the use of commercial alcalase to generate short bioactive peptides. Some research reveals that alcalase treatment can more efficiently increase the metal-binding characteristics of defatted wheat germ protein compared to flavorzyme, papain, neutrase, and protamex^15,24^. Several parameters can influence the metal-binding capabilities of hydrolyzed protein, including the degree of hydrolysis (DH) and the side chains of amino acid residues. In our previous investigation of the relationship between DH and calcium binding activity which occurred in a competition assay against calcium phosphate precipitation, we discovered that the best condition with the maximum DH had a calcium binding activity of 60.39 ± 1.545% with a final protein content of 0.1455 ± 0.0015 mg/mL^23^. This result was consistent with a study of Ke et al.^25^ who discovered that DH had a significant impact on zinc binding activity. Proper hydrolysis may expose specific groups which enhance calcium coordination^26^. Consequently, the molecular masses and side chains of the amino acid residue of DLBS were examined and debated further.

### Calcium-binding peptide purification

**Figure 1:**
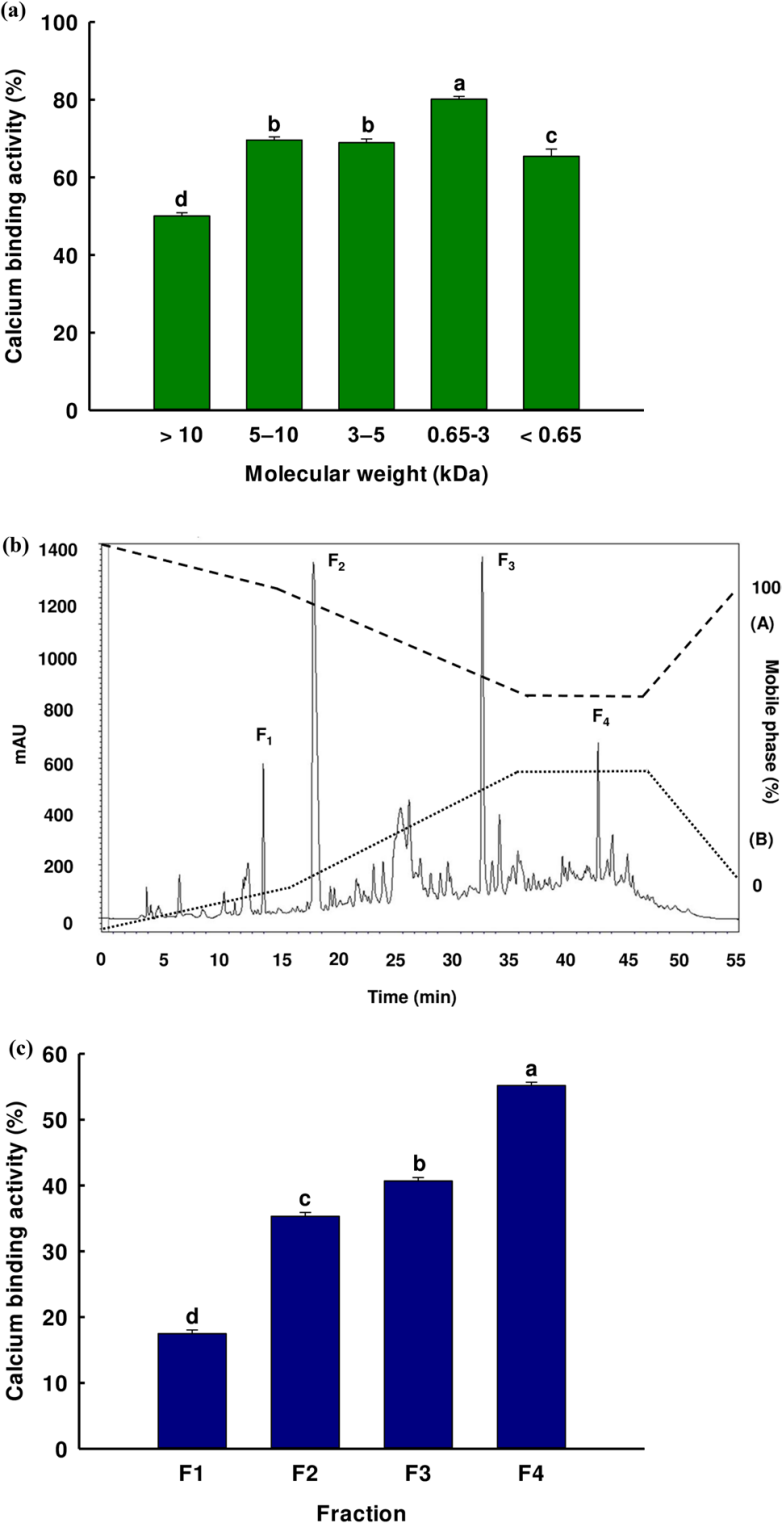
(**a**) Percentage of calcium-binding activity for each of the five fractions of the Alcalase hydrolysate following ultrafiltration; (**b**) Fractions obtained via RP-HPLC and the calcium-binding activity of the MWCO 3–0.65 kDa fraction; (**c**) calcium-binding activity of the four fractions (F_1_, F_2_, F_3_, and F_4_) separated via RP-HPLC. The values are shown as percentage of calcium-binding activity ± SD, and all testing was carried out in triplicate with a same protein concentration. Where the means is shown using a different letter, this is indicative of a significant difference in Duncan’s multiple range test (*p* < 0.05).

In this study, the DLBS protein hydrolysates first underwent purification via ultrafiltration before the peptides were classified by their MWCO. This is important because the chelating abilities of the peptides may be dependent upon molecular weight. Previous studies suggest that higher binding activity is a feature of peptides which have a lower molecular weight, as in the case of one particular peptide obtained from wheat germ protein hydrolysates (579.34 Da)^17^ and another peptide obtained from the Schizochytrium protein (291.15 Da and 328.17 Da)^27^. Figure 1a presents the results, indicating that calcium-binding activity increased as the MWCO decreased. The sample measuring 3–0.65 kDa showed the highest activity level. This stood in contrast to the case of soybean protein hydrolysate, whose calcium-binding activity declined with a lower MWCO^28^. This occurrence matches earlier studies linking peptide size with binding activity. Accordingly, the best ligand binding with calcium ions will result from an ideal size, which is neither too large nor too small^16,28,29^. After using the ultrafiltration technique, we found that the greatest calcium-binding activity peptide was presented in the 3–0.65 kDa fraction.

To study the effects of structure on the activities of calcium-binding peptides, amino acid sequences were first identified after separation. RP-HPLC was then used for the calcium-binding peptide and protein selection process. Separation via RP-HPLC is based upon the hydrophobicity of the solute, whereby a solute with high polarity will have correspondingly minimized hydrophobicity, and will therefore be less likely to combine with stationary phases. The active fraction with molecular weight 3–0.65 kDa was selected to be lyophilized and subsequently dissolved in distilled water before loading into the C18 RP-HPLC. This fraction was then further separated to create four new fractions: F_1_ (14.80 min); F_2_ (19.35 min); F_3_ (34.08 min); and F_4_ (42.85 min). Figure 1b illustrates the chromatographic profile. The fractions were then pooled to evaluate calcium binding activity, as shown in Fig. 1c. The greatest calcium-binding activity was exhibited by the F_4_ fraction. While this F_4_ fraction resulting from the RP-HPLC process had a lower activity level than the 3–0.65 kDa fraction resulting from the ultrafiltration stage, the activity of the 3–0.65 kDa fraction resulted from the activity of its combined peptides within the fraction. The peptide within the F_4_ fraction was then identified. It could be suggested that the calcium binding activity of the 3–0.65 kDa fraction occurred from the combined activity of peptides in its fraction. After finishing the RP-HPLC process, some active peptides may be eliminated from this fraction and the calcium binding activity was reduced. The peptide within the F_4_ fraction was then identified.

### Calcium-binding peptide identification

**Figure 2:**
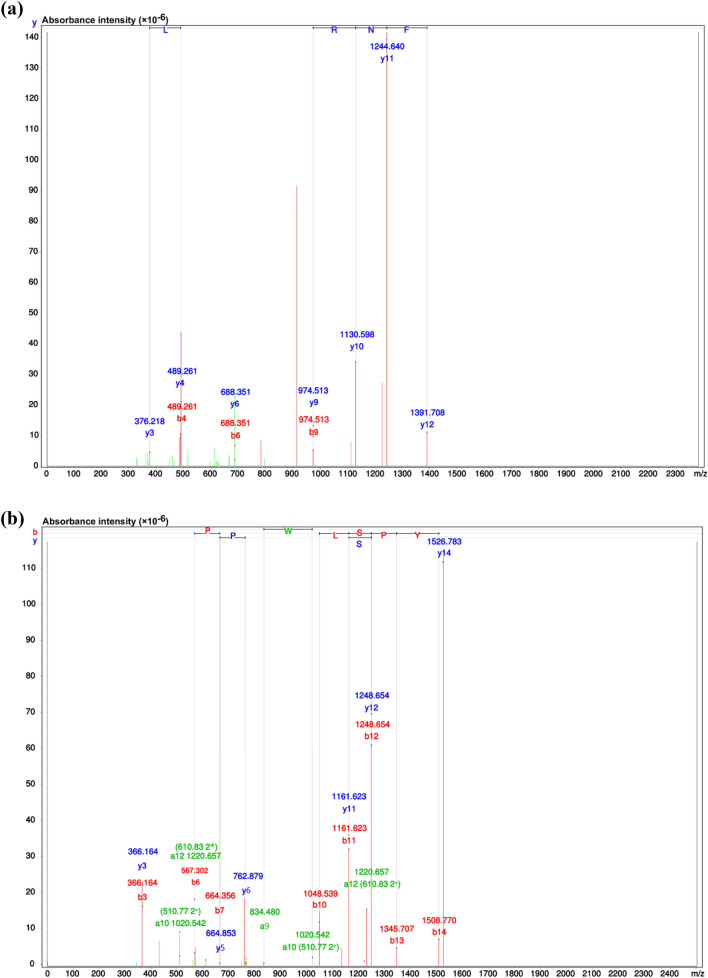
The MS/MS spectrum analysis and details of the amino acid sequence of the F_4_ fraction calcium-binding peptide obtained from the RP-HPLC process: (**a**) Basil-1, and (**b**) Basil-2.

Identification of the peptide in the F_4_ fraction after RP-HPLC was performed via mass spectrometry with de novo sequencing applied for data analysis. The findings indicate the presence of a pair of peptides: AFNRAKSKALNEN; Basil-1, (Fig. 2a) and YDSSGGPTPWLSPY; Basil-2 (Fig. 2b). Confirmation of the amino acid sequences these peptides was conducted by matching with Ocimum’s sequence using the National Center for Biotechnology Information (NCBI) database. Basil-1 exhibited a 100% (4/4) match to an amino acid sequence in the NADH dehydrogenase subunit F of *O. americanum* (SwissProt accession number AFP73817) as well as MAP-kinase of *O. bacillicum* (SwissProt accession number AMR58300.1). Meanwhile, the similarity exhibited by the Basil-2 sequence also represented a 100% (4/4) match with the amino acid sequence of NADH dehydrogenase subunit F, but there was a slight difference with the SwissProt accession number AFP73817.1. Furthermore, certain parts of the amino acid in Basil-2 show 100% (4/4) similarity to an amino acid sequence found in the cinnamyl alcohol dehydrogenase sequence of *O. tenuiflorum* (SwissProt accession number ADO16245.1).

Table 1 presents the characteristics of the two DLBS peptides, including water solubility which was obtained via the Innovagen server. The water solubility of Basil-1 was good but for Basil-2 it was poor. This finding matches the results from an in vitro study where both peptides were shown to be soluble when immersed in water, although Basil-2 dissolved much more slowly. Toxicity was analyzed via the ToxinPred server, with non-toxicity reported. Finally, sensory qualities were assessed via the BIOPEP-UWM server. In the case of Basil-1, the reported flavors from the amino acids in the sequence were listed as umami, sweet, bitter, sour, astringent, bitterness suppressing, and salt enhancing. Meanwhile, Basil-2 can be described as umami, sweet, bitter, sour, and salty. The identified peptides were then synthesized and tested to determine the calcium-binding activity; for Basil-1, the calcium-binding activity was 38.62 ± 1.33%, while for Basil-2 the activity slightly increased in comparison at 42.19 ± 2.27%, although the difference was not determined to be of significance (*P* > 0.05). It is commonly reported that the activity of short peptides exceeds that of long peptides, but this would imply that Basil-1which has a lower amino acid content than Basil-2 would show a higher level of activity, which was not the case. This result might be explained as a consequence of other effects such as the type or position of the amino acids within the sequence, or the influence of the net charge of the peptide^30,31^.

**Table 1:** Properties of Basil-1 and Basil-2.

Synthesized peptides	Water solubility*	Toxicity profile (SVM score)**	Sensory characteristics***
AFNRAKSKALNEN (Basil-1)	Good	Non-Toxic (−0.97)	Umami: E Bitter: R, F, AF, L, K Sweet: K, A Sour: E, K Astringent: K Bitterness suppressing: R, K Salt enhancer: RA
YDSSGGPTPWLSPY (Basil-2)	Poor	Non-Toxic (−0.42)	Umami: D Bitter: P, GP, GGP, L, WL, W, PY Sweet: G, P Sour: D Salty: D

*Innovagen server is the source of data on peptide solubility (www.innovagen.com/proteomics-tools).

**Peptide toxicity analysis is carried out via the ToxinPred server (http://crdd.osdd.net/raghava/toxinpred/).

***BIOPEP-UWM website (http://www.uwm.edu.pl/biochemia/index.php/pl/biopep) facilitates the sensory characterization of peptides.

Earlier research demonstrates that amino acid residues, and in particular their sequences and composition, can have a critical effect on the peptide in terms of its calcium-binding abilities^15,32^. It has been suggested that chelation with calcium ions may arise for amino, carboxyl, and phosphoric groups^12,33^. Additionally, the hydrophobic amino acid residue in the sequence was investigated for its interaction with calcium ions. It was noted that that the presence of Leu at the C- and N- terminals and in the peptide sequence leads to superior binding of metal ions compared to Leu occurring at both the C- and N- terminals. Since Leu appears in the Basil-1 and Basil-2 sequences, this may lead to improved calcium-binding activity in those particular peptides. The R group of Leu has a carbon atom which can interact with the calcium ions to create carbon–calcium bonds^16^. Further investigations were conducted on acidic amino acids and their correlation with calcium binding, and it was shown that as the acidic amino acid content increases in a peptide, the calcium-binding activity also increases. This may be due to the carboxyl groups in the acidic amino acids which provide both an oxygen atom and a suitable binding site^34–37^. In general, the interactions of metal ions and ligands occur in line with acid–base theory. The dissociation of a hydrogen ion from the ligand acid groups leads to the metal ion and the ligand combining. A significant number of peptides with metal-chelating properties have been isolated from hydrolysates, a majority of which are rich in Asp and Glu. Potential binding sites which may have a role in the coordination of chelation complexes include the COO- groups which are negatively charged as well as the NH_2_ or NH groups of amido bonds^10^. Asp and Glu amino acid residues can also be found in calcium-binding peptides which are obtained from the protein hydrolysates of cheese whey^34^, hoki (*Johnius belengerii*) bone^35^, and porcine blood plasma^37^. In this research, Basil-1 and Basil-2 contained Asp and Glu acidic amino acids. In addition, Ser, Arg, and Lys appeared in the peptide sequence and could have been involved in the calcium chelation due to the combination of calcium ions with various C = O bonds, C–OH bonds, NH_2_ bonds, and C = NH bonds. Many recent studies have sought to identify and isolate the calcium-binding peptides as well as to examine the particular amino acids and groups which are involved in the process of calcium binding.

### Fluorescence assay

**Figure 3:**
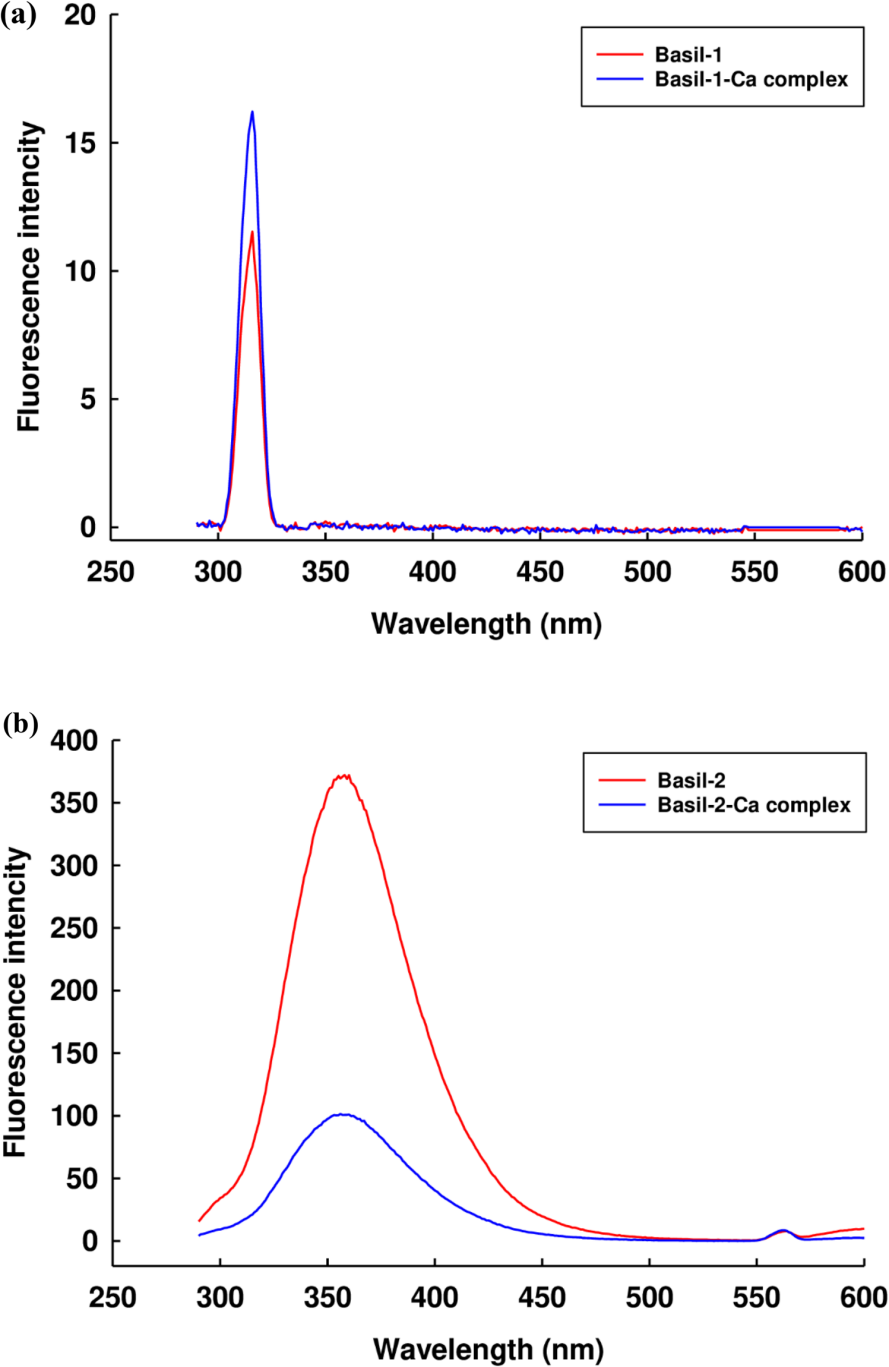
The fluorescence spectra of: (**a**) Basil-1 and the Basil-1-calcium complex; (**b**) Basil-2 and the Basil-2-calcium complex.

It was possible for the aromatic groups within the amino acid structure to produce endogenous fluorescence at suitable excitation wavelengths due to the presence of Phe, Tyr, and Typ. For this reason, structural transformations which arise as the peptide undergoes calcium binding could be detected via examination of the fluorescence spectrum^38^. The maximum absorption peak was reported at 315 nm, although the binding of calcium ions with the Basil-1 structure then resulted in increased intensity as the peak made a shift to 316 nm, as shown in Fig. 3a. This outcome concurs with earlier accounts of the reaction taking place between β-lactoglobulin hydrolysate and iron ions, whereby the maximum peak shifted to a lower absorption peak during chelation, with a change in intensity^39^. The maximum absorption peak for Basil-2 occurred at 358 nm. Following chelation with calcium ions, this value shifted to 356 nm while the intensity also declined, as can be seen in Fig. 3b. Knappskog et al.^40^ and Miranda et al.^41^ reported that during the metal-binding reaction, three aromatic amino acids moved to the peptide structure surface, which caused the alteration of the maximum absorption peak. Uppal et al.^42^ argued that fluorescence quenching might be a consequence of a heightened concentration of metal ions, as indicated by a decline in the intensity. Zhou et al.^39^ produced similar results, observing that fluorescence quenching occurred as the calcium ions entered into combination with the calcium-chelating peptide. Furthermore, Wu et al.^43^ demonstrated that the reduction in the intensity of fluorescence commonly resulted from peptide folding as ferrous ions underwent chelation with peptides from sturgeon proteins, since during the folding process, those ferrous ions closed to tryptophan residues. The findings might suggest that the binding process between these peptides and the calcium ions could cause a blue shift at the maximum fluorescence peak, and is indicative of a clear difference between the original peptide and the new calcium-peptide complex.

### FTIR measurement

**Figure 4:**
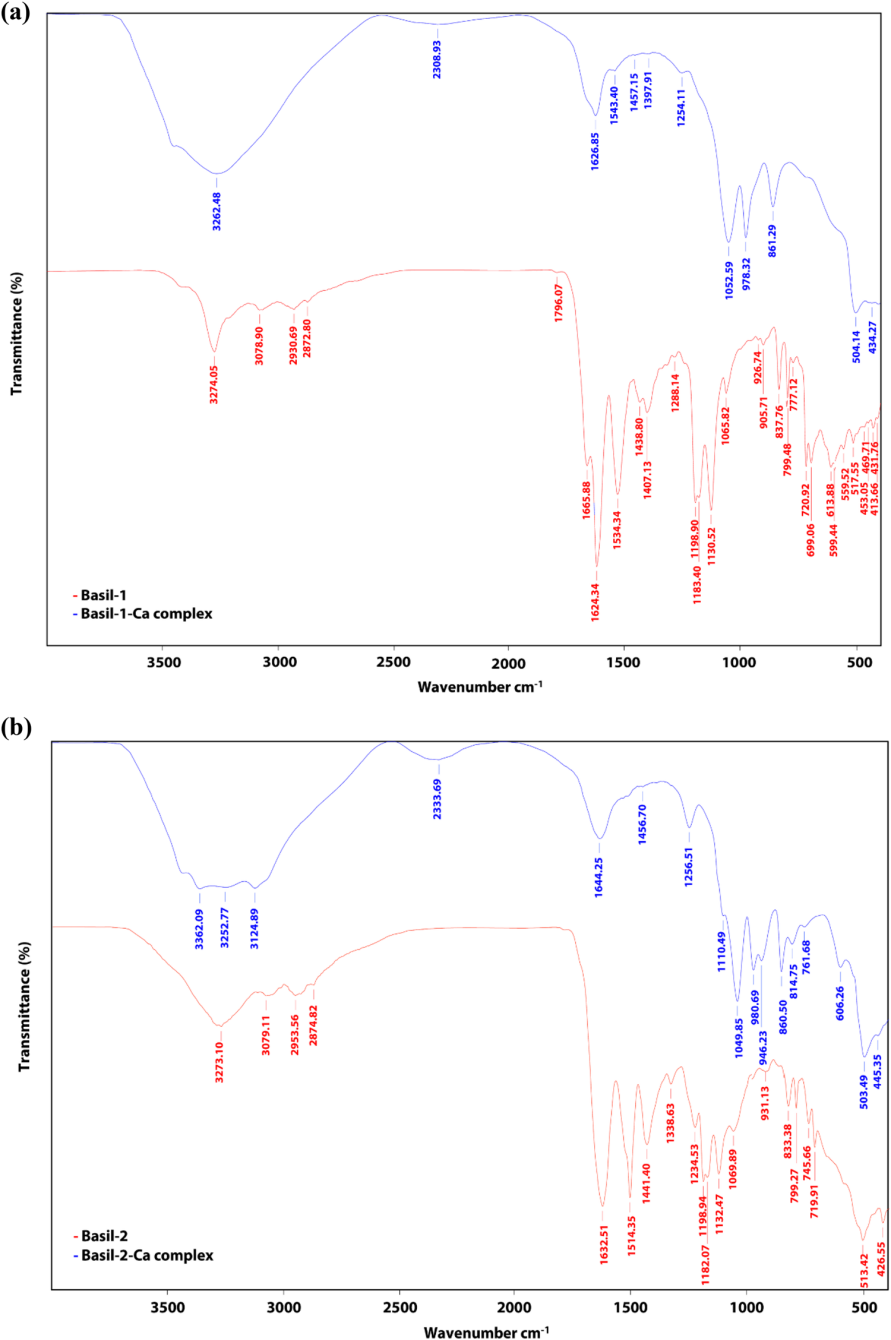
The FTIR spectra of: (**a**) Basil-1 and the Basil-1-calcium complex; (**b**) Basil-2 and the Basil-2-calcium complex.

FTIR allows the detection of peptide changes after calcium binding occurs. FTIR works by identifying the functional groups which occur within the sequence. Metal ions will usually form bonds with carbonyl or carboxyl groups or the amide group of amino acid residues found within the peptide segment. Accordingly, there is a shift in the calcium-peptide complex absorption peaks when compared to those of the initial peptide^39^. Figure 4a presents the different absorption peaks of the Basil-1 and Basil-1-Ca complexes. Following chelation of the Basil-1 sequence with calcium ions, there was a shift in the absorption peak for Basil-1 from 3274.05 cm^-1^ to 3262.48 cm^-1^, while three of the peaks completely disappeared. The wavenumbers represent the amide A band (3500–2800 cm^-1^), since it is possible for the nitrogen atom in the N–H bonds to coordinate with the calcium ions as an electron pair is offered^44,45^. The amide-I and amide-II vibrations were of particular significance; C = O bond stretching leads to the amide-I vibration (1700–1600 cm^-1^), while C–N bond stretching and N–H bond deformation lead to the amide-II vibration (1600–1500 cm^-1^)^46,47^. Moreover, there was shift in the amide-I band of Basil-1 from 1624.34 to 1626.85 cm^-1^, and in the amide-II band of Basil-1 from 1534.34 to 1543.40 cm^-1^ after chelation with calcium ions. The amide-I and amide-II bands of Basil-1 decreased sharply in absorption intensity once the complex had been formed with the calcium ions. A pair of small peaks arising in the range of 1500 cm^-1^–1400 cm^-1^ appeared after chelation with calcium ions, potentially due to the oxygen atom in the carboxylate group which then encounters the calcium ions to form COO–Ca. The final wavenumber which is seen from 800 to 500 cm^-1^ may result from the O–Ca vibrational mode arising when metal ions and ligand atoms (nitrogen, oxygen, sulfur) combine^33,36^ which can be observed as the wavenumber alters from 517.55 to 504.14 cm^-1^.

Figure 4b presents the contrasting absorption peaks for Basil-2 and the Basil-2-Ca complex. For amide-I, there were four peaks in the band wavenumber of the original peptide, but the first of these absorption peaks at 3273.10 cm^-1^ shifted to a lower frequency region around 3252.77 cm^-1^ and exhibited some different intensities, whereas the second peak at 3079.11 cm^-1^ shifted to a much greater absorption wavenumber of 3124.89 cm^-1^, while the other peaks disappeared as a consequence of the interaction between the calcium ions and the nitrogen atom from the N–H bonds. In the amide-I band, there was a shift in the absorption peak from 1632.51 to 1644.25 cm^-1^. However, as a consequence of the coordinate binding involving calcium ions and the C–N and N–H bonds of the amide-II vibration, the absorption peak at 1514.35 cm^-1^ disappeared, suggesting the creation of C–Ca and N–Ca within the amide-II wavenumber. Furthermore, there is an influence upon the absorption peak of the original peptide at 1441.40 cm^-1^ exerted by the –COO–Ca combination, which induces a shift to 1456.70 cm^-1^ and a change in the intensity. In addition, there were two other Basil-2 absorption peaks at 745.66 cm^-1^ and 513.42 cm^-1^ which underwent shifts to 761.63 cm^-1^ and 503.49 cm^-1^ as the ligand atom interacted with the calcium ion. It can thus be argued that there was interaction between the calcium ions and the C = O bonds, –COO– bonds, N–H bonds, and C–N bonds as the peptide calcium complex was formed. The chelation produces a different absorption peak for the peptide-calcium complex when compared to the original peptide, and thus the new peaks confirm the existence of a new substance. This matches the findings for FTIR spectra of metal ions chelated with original peptides, and was confirmed in the works of Zhang et al.^33^ and Chen et al.^48^, while Wang et al.^17^ observed that when metal ions and peptide functional groups interact, the outcome can be a wider or weaker absorption peak, or in some cases it disappears.

Presently, a computer simulation method was used to explore the binding position and the interaction between ligand and receptor. Numerous studies have argued that the binding of calcium and peptides depends upon the particular groups that are found in the peptide sequence. The groups that most frequently appear in the literature in the context of calcium binding are phosphate and carboxyl groups^49,50^. In the human diet, milk casein from cows and egg yolk casein contain phosphorylated groups, including CPPs and phosvitin phosphopeptides (PPPs), which are able to aggregate calcium via highly polar acidic motifs. These phosphorylated groups are ideal for calcium binding because of their phosphoserine residues, which produce amorphous Ca_3_(PO_4_)_2_ nanoparticles when exposed to calcium^51^. Various peptides which do not feature such phosphorylated residues are also known to bind calcium through the presence of the carboxyl group, Glu, and Asp. These include peptides derived from whey proteins^52^, bovine serum proteins^53^, soybean proteins^54^, and tilapia scale proteins^26^. The reason for the success of carboxylic groups in creating these complexes lies in the presence of carbonyl oxygen, which offers a non-bonding free electron pair which then performs the chelation of metal ions that have empty electron shells. In addition, sulfhydryl groups, the nitrogen-rich group of the His imidazole, hydrophilic groups, and negatively charged groups play significant roles in calcium-binding^55^. This part might be supported by the result of the FTIR assay because the prediction of calcium binding sites was obtained at carbonyl group, carboxyl group, C–H bond, C–C bond, and N–H bond which are correlated with the change in FTIR spectra when the peptide is formed into the calcium-peptide complex. Otherwise, some metal ions have been reported to interact with these function groups, which has a major role in metal ion chelation^56,57^.

### Calcium uptake and cell viability of Caco-2 cells

**Figure 5:**
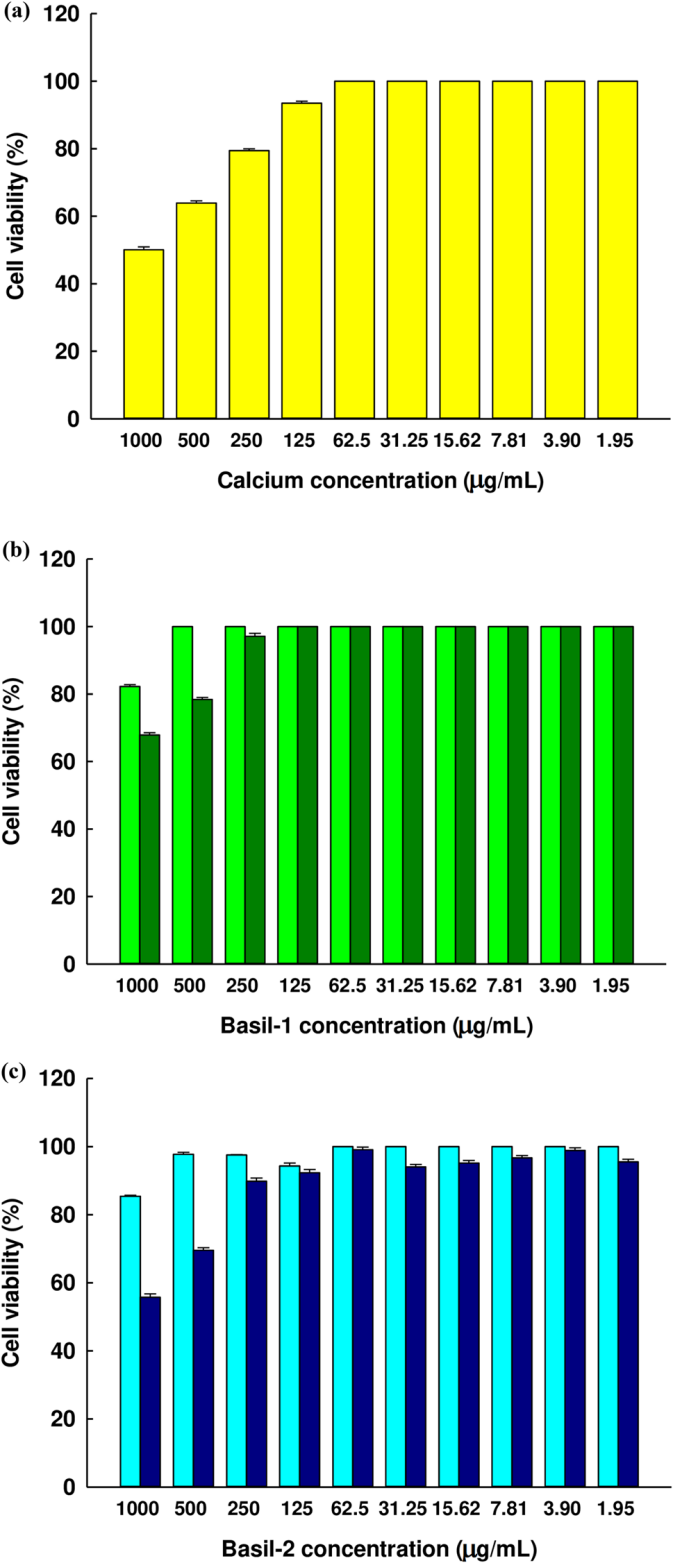
The viability of the Caco-2 cells following treatment at varying concentrations of (**a**) CaCl_2_ solution (), (**b**) Basil-1 () and Basil-1-calcium complex (), and (**c**) Basil-2 () and Basil-2-calcium complex () The graph shows the mean ± SE (n = 3).

Human Caco-2 cells are adenocarcinoma cells found in the intestines and offer biochemical and morphological properties similar to enterocytes. As such, they have been shown to play a key role in studies of calcium absorption along with other minerals when examined *in vitro*^28,58^. Accordingly, the researchers sought to investigate the influence of Basil-1 and Basil-2 on the calcium uptake of Caco-2 cell monolayers. Initially, the MTT assay was performed to determine the potential range of concentrations for the samples. Figure 5a-c shows cell viability over 90% following treatment with original peptides, peptide-calcium complexes, and calcium chlorine solution at various concentrations.

**Figure 6:**
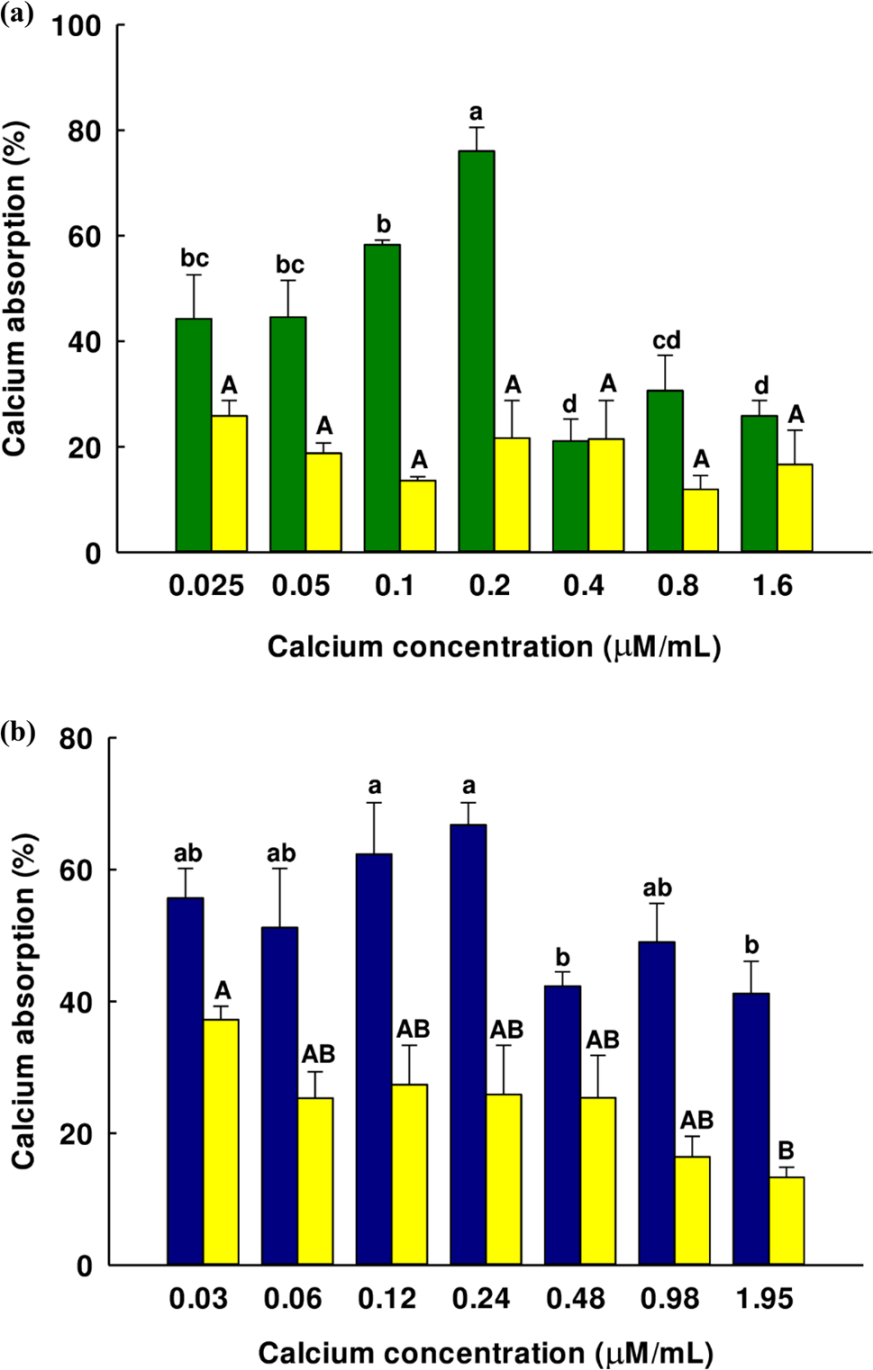
(**a**) Effects of Basil-1-calcium complex (), CaCl_2_ () solution and (**b**) Basil-2-calcium complex () on the uptake of calcium in Caco-2 cell monolayers at different CaCl_2_ concentrations. The values are shown as percentage of calcium-binding activity ± SD, while all testing was carried out in triplicate. Where the mean is shown with a different letter, this indicates a significant difference in Duncan’s multiple range test (*p* < 0.05).

Based on the MTT results, the concentration of Basil-1-Ca complex and Basil-2-Ca complex at 250 µg/mL exhibited 0.2 µg/mL and 0.24 µg/mL calcium concentration, respectively. These calcium concentrations in the complexes were the maximum concentrations that did not cause toxicity and were selected to determine calcium absorption. Caco-2 cells were cultured with varying quantities of calcium ions from the peptide-calcium complex, while free calcium from CaCl_2_ was used as a comparison. Figure 6 shows the effect of free calcium ions and calcium ions from the complex in the Caco-2 cell monolayer model. The calcium ions in the Basil-1-Ca complex were shown to have a raised absorption when the calcium concentration in the complex was increased until it reached 0.2 µg/mL, which was the maximum absorption (Fig. 6a). At this concentration, Caco-2 cells absorb 77% of the calcium ion when compared to the control (cells without calcium ion). Moreover, the uptake-enhanced effects of the Basil-1-Ca complex were over three times greater than those of CaCl_2_ when the calcium concentration was maintained fixed at 0.2 µg/mL. In contrast to the results obtained with free calcium ions from calcium chloride solution, there was no significant difference in the results obtained with calcium uptake when the concentrations of free calcium ions from calcium chloride solution were varied (*P* < 0.05). Although the range of calcium chloride solutions in the calcium absorption study did not inhibit the growth of Caco-2 cells, all Caco-2 cells were active cells that had a maximum calcium absorption rate, so the increase of free calcium ions did not promote calcium absorption.

When considering calcium ions transported from the Basil-2-Ca complex, absorption increased slightly as the calcium concentration rose from 0.03 to 0.24 µg/mL, leading to a maximum absorption of 66.78% at that final concentration of 0.24 µg/mL. In contrast, the maximum free calcium ion absorption from calcium chloride solution occurred at 0.03 µg/mL, differing significantly from the absorption at 1.95 µg/mL of calcium but with no significant difference from other concentrations (*P* < 0.05), as shown in Fig. 6b. This result indicates that the calcium uptake efficiency of the Basil-2-Ca complex was also remarkably higher than free calcium. In the past, research has been published on the promotion of calcium uptake from calcium-peptide complexes, such as the fish scales protein hydrolysate-calcium complex (FSPH-ca)^59^_,_ desalted duck egg white peptide-calcium (DPs-calcium)^60^, Ca-casein phosphopeptides (Ca-CPPs)^14^, and bovine serum protein hydrolysate-calcium complex (BSP-ca)^53^ by acting as calcium carriers and affecting the calcium receptor on the membrane of intestinal cells to promote calcium uptake.

Calcium ions are normally transported into cells via both passive and active transportation. Earlier studies have found that the chelation of calcium ions with peptides or protein hydrolysates may support the transportation of calcium since the peptide is able to carry the calcium and overcome the obstacle of the plasma membrane, thus allowing the calcium ions to reach the Caco-2 cells^13,28,61^. It may be possible for the peptide-calcium complex to prevent the precipitation of calcium in the intestines, thus boosting calcium ion concentrations in the extracellular cytoplasm. This can effectively diffuse calcium or allow intracellular passive transportation. Earlier research indicates that an increase in the uptake of calcium correlates if Glu and Asp are present within the sequence^62^. Moreover, the basic amino acids (Lys, His, and Arg) are positively charged and can therefore influence calcium transportation as they interact with the cell membrane and its negative charge^63^. For this reason, it is possible that the presence Arg, Glu, and Lys in the Basil-1-Ca complex and of Asp in the Basil-2-Ca complex could support the transportation of calcium into Caco-2 cells. It may further be possible to conclude that calcium absorption is promoted by Basil-1-Ca and the Basil-1-Ca complex through the prevention of calcium precipitation, which leads to increased transportation of calcium through the cells of the intestines.

## Materials and methods

### Chemicals and biomaterials

The Thai lemon basil seeds were obtained for this study from a farm located 50 m above sea level in Si Samrong District, Sukhothai Province, with permission from Royal Thai Seeds Co., Ltd. The sample was harvested in December 2018 at an age of 120 days. The sample of seeds was then dried in air and placed in storage in darkness at a temperature of 4 °C. All chemicals used in this research were of analytical grade and were supplied as follows: Novo Nordisk (Bagsverd, Denmark) supplied the Alcalase 2.4 L which is a microbial protease of *Bacillus licheniformis*. This product was also stored at 4 °C. Sigma-Aldrich (St. Louis, MO, USA) supplied the 99% trifluoroacetic acid (TFA) and bovine serum albumin (BSA), while Merck (Darmstadt, Germany) provided disodium hydrogen phosphate (Na_2_HPO_4_), calcium chloride (CaCl_2_), and monosodium dihydrogen orthophosphate (NaH_2_PO_4_), all of which were placed into storage at room temperature. Finally, Thermo Fisher Scientific (MA, USA) provided Fluo-3 AM (Fluorescent calcium indicator) which was stored until use at −20 °C.

### DLBS protein hydrolysate preparation

A modified version of the technique proposed by Sakdasri et al.^64^ was employed to prepare the DLBS, while the protein hydrolysates of DLBS underwent preparation using the optimal proteolysis parameters, which had previously been determined to involve a hydrolysis time of 103 min, a temperature of 55℃, and an enzyme-to-substrate ratio of 7.0% w/v^23^. The reaction was then halted by increasing the temperature to 90 °C for 20 min. Then the supernatant was removed and centrifuged for 30 min at 15,900×*g* at 4 °C, before being placed into storage at −20 °C. Bradford’s method was employed to determine the concentration of the protein in the supernatant, using BSA as the protein standard^65^.

### Activity for calcium-binding

The calcium-binding activity was evaluated in line with a modified version of the approach proposed by Cai et al.^27^ The addition of 1 mL of 0.5 mg/mL protein hydrolysates to 1 mL of 9 mM CaCl_2_ and 2 mL of 200 mM sodium phosphate buffer at a pH value of 7.5 created a mixture which supported competitive conditions arising between the protein hydrolysates and phosphate ions. During the 2-h incubation period, the binding reaction commenced at 37 °C and 150 rpm. Centrifugation was then employed at 15,900×*g* rpm for 10 min in order to extract and eliminate the insoluble calcium phosphate salts, whereupon the remaining soluble calcium was evaluated using atomic absorption spectrophotometry (ET-AAS, VARIAN AA 280 FS, Agilent, USA) under the atomic absorption spectrometric method 3500-Ca, while protein concentration was 0.1455 ± 0.0015 mg/mL^23^. Equation (1) given below represents the activity for calcium binding:

1 $$ {\text{Calcium-binding}}\;{\text{activity}}\,\left( \% \right) = C_{{{\text{CP}}}} /C_{t} \times 100\% $$

where C_CP_ indicates the calcium content within the calcium-protein hydrolysate/peptide complex (mg/L), and C_t_ indicates the overall calcium content within the solution (mg/L).

## Purification of the peptides

### Ultrafiltration

Ultrafiltration of the DLBS protein hydrolysates was carried out via fractionation using a series of four membranes for which the molecular weight cut-offs (MWCO) were set to 10 kDa, 5 kDa, 3 kDa, and 0.65 kDa (Minimate TFF Capsule, Pall Corporation, USA). In the first step, Whatman No.1 filter paper was used to eliminate the rugged precipitate from the hydrolysates, whereupon the solution underwent ultrafiltration in which the largest membrane was 10 kDa MWCO. This produced an initial retentate where the MW exceeded 10 kDa, while the filtrate was less than 10 kDa. Analysis of the retentate was performed, and then the process was repeated using successively smaller filters of 5 kDa, 3 kDa, and 0.65 kDa, which resulted in the five fractions: > 10 kDa, 5–10 kDa, 3–5 kDa, 0.65–3 kDa, and < 0.65 kDa. These fractions were then lyophilized prior to storage at −20 °C until further use. An evaluation of the calcium-binding capacities was carried out in order that the fraction producing the greatest capacity could then undergo further purification through reversed-phase high performance liquid chromatography (RP-HPLC).

### RP-HPLC

The fraction from the initial ultrafiltration step which offered the best calcium-binding activity was chosen for the next stage involving a 0.45 µm filter (Whatman, GE, Buckinghamshire, UK), followed by separation via RP-HPLC using a C18 column (Shimpak, 250 × 46 mm, Luna SU; Phenomenex, Torrance, CA, USA). The quantity injected was 100 µL, and the protein concentration in the sample was ⁓1.0 µg protein/mL with a flow rate held constant at 0.7 mL/min. To elute different fractions at different times, the ratios of solution A (0.1% (v/v) TFA) to solution B (70% (v/v) acetonitrile in 0.05% (v/v) TFA) were varied accordingly. ChromQuest Software Thermo Fisher Scientific (MA, USA) was employed to carry out the chromatographic analysis, with all peptide fractions undergoing detection at 280 nm. The calcium-binding activity of all the fractions obtained was assessed, and the fractions were then lyophilized in preparation for further examination.

### Identification and synthesis of peptides

Identification of the peptide contained within the RP-HPLC fraction offering the greatest calcium-binding activity was achieved through the use of an electrospray ionization and a Q-TOF mass spectrometer (ESI; model Amazon SL, Bruker, Germany), whereupon the data were subsequently analyzed using a de novo sequencing approach. The peptide characteristics were then further assessed using the ToxinPred server (http://crdd.osdd.net/raghava/toxinpred/), the Innovagen server (www.innovagen.com/proteomics-tools), and also the BIOPEP-UWM server (http://www.uwm.edu.pl/biochemia/index.php/pl/biopep) in order to determine the peptide toxicity and water solubility. Having identified the peptides, further synthesis was performed via Fmoc solid-phase with an Applied Biosystems Model 433A Synergy peptide synthesizer (Applied Biosystems, Foster City, CA, USA). Peptide purity was then confirmed via analytical mass spectrometry using a quadrupole ion trap Thermo Finnigan™LXQ™LC–ESI–MS (San Jose, CA, USA) linked to a Surveyor HPLC (Thermo Fisher Scientific, San Jose, CA, USA). The identified peptide sequences were Ala-Phe-Asn-Arg-Ala-Lys-Ser-Lys-Ala-Leu-Asn-Glu-Asn (AFNRAKSKALNEN; Basil-1) and Tyr-Asp-Ser-Ser-Gly-Gly-Pro-Thr-Pro-Trp-Leu-Ser-Pro-Tyr (YDSSGGPTPWLSPY; Basil-2), which offered respective molecular weights of 1462.80 Da and 1526.81 Da, along with HPLC purity of 98%. Finally, measurements of the calcium-binding capacities of these synthetic peptides were recorded via the technique applied earlier in this study.

## Calcium-peptide complex structure characterization

### Fluorescence spectroscopy

During calcium peptide binding, a conformational change in the peptide takes place, which can be measured via fluorescence spectroscopy with a Varian Cary Eclipse Fluorescence Spectrophotometer (Varian Optical Spectroscopy Instruments Mulgrave, Victoria, Australia). In this case, the excitation wavelength was 285 nm and the emission wavelength was recorded in the range of 250 nm to 400 nm. The slit width for excitation was 20 nm while for emission it was 30 nm, while the sensitivity was 1. Sample preparation was carried out in a manner identical to that of the ultraviolet spectroscopy analysis.

### Fourier transform infrared spectroscopy (FTIR)

In line with the guidelines of Nara et al.^46^ the FT-IR spectra of peptide along with its associated calcium complex were determined. In this process, the initial mixture comprising 100 mg or dry KBr and 1 mg of the freeze-dried sample was placed under infrared light for grinding, whereupon it was then pressed to form a thin disc. The disc absorbance was then measured using a wavenumber in the range of 4000 cm^-1^ to 400 cm^-1^, at a resolution of 4 cm^-1^ with scan times of 32 using a PerkinElmer Spectrum ONE FTIR Spectrometer (SpectraLab Scientific Inc., Markham, Ontario, Canada).

## The influence of the calcium-peptide complex on calcium uptake in Caco-2 cells

### Cell culture and viability

Caco-2 cells form the human intestinal cell line were obtained from ATCC (American Type Culture Collection, Teddington, United Kingdom). The cell lines were grown in a cell culture flask (Thermo Fisher Scientific (MA, USA) in Eagle’s Minimum Essential Medium (EMEM) with 10% (v/v) fetal bovine serum (FBS) at 37 °C and 5% carbon dioxide in a humid incubator, before serving as a model to represent the intestinal barrier. During the growth phase, the cell completeness was checked twice weekly while the medium was changed. Having reached 90% confluence, the cells then underwent detachment using 0.05% trypsin–EDTA solution, before seeding in 96-well cell culture plates in which the selected cell density was 1 × 10^4^ cells/well. MTT assay was used to evaluate the cell toxicity while the experiments varied the concentrations of calcium-peptide complexes, free peptides, and calcium solution. Following incubation for a further 24 h, the medium was removed in order for 100 µL of 0.5 mg/mL MTT solution to be introduced to each well, whereupon the incubation was continued for 2 more hours. The treated medium was then discarded before the addition of 100 µL of dimethyl sulfoxide in order to solubilize the formazan crystals which had been produced. A spectrophotometer was used to assess the optical density of the samples at 490 nm. Finally, the percentage cell viability was calculated by comparing the quantity of surviving cells in the test wells to the values obtained in the control wells.

### Calcium uptake

The calcium uptake unto Caco-2 cells was assessed using a slightly modified version of Lin’s technique^59^. Initially, a 96-well black polystyrene microplate was used for the seeding of the Caco-2 cells at concentrations of 6 × 10^4^ cells/well prior to incubation for 24 h at a temperature of 37 °C, under conditions of 5% carbon dioxide. This assay had 2 groups first was a group with fluorescent treatment and second was a blank group without fluorescence. The cells in each group were treated with samples consisted of calcium-peptide complexes, and a calcium solution with an equal concentration of calcium, prior to incubation once again under identical conditions for a further 2 h, while cells without sample as a control. All cells were then washed using 200 µL of sterile phosphate buffer solution (PBS) with the procedure performed in triplicate. The first group followed by the addition of 100 µL of 2.5 µM Fluo-3-AM, while the one was added by 100 µL of PBS for a further 1 h. Washing of the excess fluorescence dye was again carried out three times using PBS before adding 100 µL of PBS prior to taking measurements. Fluorescence detection was performed with an excitation wavelength set to 485 nm while the emission wavelength was set to 525 nm. This procedure involved the use of a fluorescence spectrophotometer (Synergy HT, BioTek, VT, USA). As the quantity of calcium ions in the Caco-2 cell increases, this will be reflected in the fluorescence score and can be compared to an untreated cell sample as the control. The percentage of calcium uptake was calculated from this equation.

2 $$ {\text{Calcium}}\;{\text{uptake}}\,\left( \% \right) = \left[ {\left( {{\rm C}_{{\rm pf}} - {\rm C}_{{\rm p}} } \right) - \left( {{\rm C}_{{\rm f}} - {\rm C}} \right)} \right]/\left( {{\rm C}_{{\rm f}} - {\rm C}} \right) \times 100\% $$

where C_pf_ was the fluorescence score from calcium content in calcium-peptide complex with Fluo-3-AM in the cells, C_p_ was the fluorescence score from calcium content in calcium-peptide complex without Fluo-3-AM in the cells, C_f_ was the fluorescence score from calcium contend in the cells with Fluo-3-AM, and C was a fluorescence score from calcium content in the cells without Fluo-3-AM.

### Statistical analysis

The experiments were all performed in triplicate, with findings presented in the form of mean ± standard deviation (SD). The data analysis procedures made use of SPSS version 11.5 (SPSS Inc., Chicago, IL, USA), while sample differences were evaluated using Duncan’s multiple range test and ANOVA, with a significance level set to *P* < 0.05. The author confirms that all methods were performed in accordance with the relevant guidelines and regulations by the “IUCN Policy Statement on Research Involving Species at Risk of Extinction” and the “Convention on the Trade in Endangered Species of Wild Fauna and Flora”.

## Conclusion

Using the approach described, DLBS protein was employed as a source of chelating peptides. The two novel peptides, Basil-1 and Basil-2, were separated from the protein hydrolysate of DLBS. The findings revealed that the calcium-binding activity was strongly affected by both the molecular mass of the peptide and its amino acid composition. The chelating reaction makes use of the oxygen atoms found in the carbonyl groups, and the nitrogen atoms found in the amino groups. Our study made use of synthesized peptides which are commercially available. We then examined the calcium chelating activity of these peptides and the influence they can exert upon the uptake of calcium in Caco-2 cell monolayers. It can be concluded that DLBS protein serves as an excellent source from which bioactive peptides can be produced, and then used in functional foods. The bioavailability, however, will require evaluation through further studies involving both in vitro and in vivo assays.

## Data Availability

All data generated or analyzed during this study are included in this published article.

## References

[1] Anderson JJ (2001). Calcium requirements during adolescence to maximize bone health. J. Am. Coll. Nutr..

[2] Cashman KD (2002). Calcium intake, calcium bioavailability and bone health. Br. J. Nutr..

[3] Guéguen L, Pointillart A (2000). The bioavailability of dietary calcium. J. Am. Coll. Nutr..

[4] Lane NE (2006). Epidemiology, etiology, and diagnosis of osteoporosis. Am. J. Obstet. Gynecol..

[5] Adluri RS, Zhan L, Bagchi M, Maulik N, Maulik G (2010). Comparative effects of a novel plant-based calcium supplement with two common calcium salts on proliferation and mineralization in human osteoblast cells. Mol. Cell Biochem..

[6] Li K, Wang XF, Li DY, Chen YC, Zhao LJ, Liu XG, Guo YF, Shen J, Lin X, Deng J, Zhou R, Deng HW (2018). The good, the bad, and the ugly of calcium supplementation: a review of calcium intake on human health. Clin. Interv. Aging..

[7] Milner-White EJ, Russell MJ (2005). Nests as sites for phosphates and iron-sulfur thiolates in the first membranes: 3 to 6 residue anion-binding motifs. Orig. Life Evol. Biosph..

[8] Donida BM, Mrak E, Gravaghi C, Villa I, Cosentino S, Zacchi E, Perego S, Rubinacci A, Fiorilli A, Tettamanti G, Ferraretto A (2009). Casein phosphopeptides promote calcium uptake and modulate the differentiation pathway in human primary osteoblast-like cells. Peptides.

[9] Walker G, Cai F, Shen P, Reynolds C, Ward B, Fone C, Honda S, Koganei M, Oda M, Reynolds E (2006). Increased remineralization of tooth enamel by milk containing added casein phosphopeptide-amorphous calcium phosphate. J. Dairy Res..

[10] Walters ME, Esfandi R, Tsopmo A (2018). Potential of food hydrolyzed proteins and peptides to chelate iron or calcium and enhance their absorption. Foods..

[11] Platel K, Srinivasan K (2016). Bioavailability of micronutrients from plant foods: an update. Crit. Rev. Food Sci. Nutr..

[12] Sun X, Sarteshnizi RA, Boachie RT, Okagu OD, Abioye RO, Pfeilsticker Neves R, Ohanenye IC, Udenigwe CC (2020). Peptide-mineral complexes: understanding their chemical interactions, bioavailability, and potential application in mitigating micronutrient deficiency. Foods..

[13] Perego S, Cosentino S, Fiorilli A, Tettamanti G, Ferraretto A (2012). Casein phosphopeptides modulate proliferation and apoptosis in HT-29 cell line through their interaction with voltage-operated L-type calcium channels. J. Nutr. Biochem..

[14] Perego S, Del Favero E, De Luca P, Dal Piaz F, Fiorilli A, Cantu L, Ferraretto A (2015). Calcium bioaccessibility and uptake by human intestinal like cells following in vitro digestion of casein phosphopeptide-calcium aggregates. Food Funct..

[15] Liu FR, Wang L, Wang R, Chen ZX (2013). Calcium-binding capacity of wheat germ protein hydrolysate and characterization of Peptide-calcium complex. J. Agric. Food Chem..

[16] Budseekoad S, Yupanqui CT, Sirinupong N, Alashi AM, Aluko RE, Youravong W (2018). Structural and functional characterization of calcium and iron-binding peptides from mung bean protein hydrolysate. J. Funct. Foods..

[17] Wang L, Ding Y, Zhang X, Li Y, Wang R, Luo X, Li Y, Li J, Chen Z (2018). Isolation of a novel calcium-binding peptide from wheat germ protein hydrolysates and the prediction for its mechanism of combination. Food Chem..

[18] Liu H, Lv Y, Xu J, Guo S (2016). Soybean peptide aggregates improved calcium binding capacity. LWT Food Sci. Technol..

[19] Salehi F, Kashaninejad M, Tadayyon A, Arabameri F (2015). Modeling of extraction process of crude polysaccharides from Basil seeds (*Ocimum basilicum* l.) as affected by process variables. J. Food Sci. Technol..

[20] Mirabolhassani SE, Rafe A, Razavi SM (2016). The influence of temperature, sucrose and lactose on dilute solution properties of basil (*Ocimum basilicum*) seed gum. Int. J. Biol. Macromol..

[21] Nazir S, Wani IA, Masoodi FA (2017). Extraction optimization of mucilage from Basil (*Ocimum basilicum* L.) seeds using response surface methodology. J. Adv. Res..

[22] Kongcharoen A, Poolex W, Wichai T, Boonsombat R (2016). Production of an antioxidative peptide from hairy basil seed waste by a recombinant *Escherichia coli*. Biotechnol. Lett..

[23] Kheeree N, Sangtanoo P, Srimongkol P, Saisavoey T, Reamtong O, Choowongkomon K, Karnchanatat A (2020). ACE inhibitory peptides derived from de-fatted lemon basil seeds: optimization, purification, identification, structure-activity relationship and molecular docking analysis. Food Funct..

[24] Zhu K-X, Wang X-P, Guo X-N (2015). Isolation and characterization of zinc- chelating peptides from wheat germ protein hydrolysates. J. Funct. Foods..

[25] Ke X, Hu X, Li L, Yang X, Chen S, Wu Y, Xue C (2021). A novel zinc-binding peptide identified from tilapia (*Oreochromis niloticus*) skin collagen and transport pathway across Caco-2 monolayers. Food Biosci..

[26] Chen D, Mu X, Huang H, Nie R, Liu Z, Zeng M (2014). Isolation of a calcium- binding peptide from tilapia scale protein hydrolysate and its calcium bioavailability in rats. J. Funct. Foods..

[27] Cai X, Lin J, Wang S (2016). Novel peptide with specific calcium-binding capacity from *Schizochytrium* sp. protein hydrolysates and calcium bioavailability in Caco-2 cells. Mar Drugs..

[28] Lv Y, Bao XL, Yang BC, Ren CG, Guo ST (2008). Effect of soluble soybean protein hydrolysate-calcium complexes on calcium uptake by Caco-2 cells. J. Food Sci..

[29] Nie R, Liu Y, Liu Z (2014). The calcium-binding activity of fish scale protein hydrolysates. J. Agric. Chem. Environ..

[30] Chaud MV, Izumi C, Nahaal Z, Shuhama T, Bianchi ML, de Freitas O (2002). Iron derivatives from casein hydrolysates as a potential source in the treatment of iron deficiency. J. Agric. Food Chem..

[31] Lv Y, Liu Q, Bao X, Tang W, Yang B, Guo S (2009). Identification and characteristics of iron-chelating peptides from soybean protein hydrolysates using IMAC-Fe^3+^. J. Agric. Food Chem..

[32] Zhao L, Cai X, Huang S, Wang S, Huang Y, Hong J, Rao P (2015). Isolation and identification of a whey protein-sourced calcium-binding tripeptide Tyr-Asp-Thr. Int. Dairy J..

[33] Zhang X, Jia Q, Li M, Liu H, Wang Q, Wu Y, Niu L, Liu Z (2021). Isolation of a novel calcium-binding peptide from phosvitin hydrolysates and the study of its calcium chelation mechanism. Food Res. Int..

[34] Kim SB, Lim JW (2004). Calcium-binding peptides derived from tryptic hydrolysates of cheese whey protein. Asian Aust. J. Animal Sci..

[35] Jung WK, Park PJ, Byun HG, Moon SH, Kim SK (2005). Preparation of hoki (*Johnius belengerii*) bone oligophosphopeptide with a high affinity to calcium by carnivorous intestine crude proteinase. Food Chem..

[36] Bao XL, Lv Y, Yang BC, Ren CG, Guo ST (2008). A study of the soluble complexes formed during calcium binding by soybean protein hydrolysates. J. Food Sci..

[37] Lee SH, Song KB (2009). Isolation of a calcium-binding peptide from enzymatic hydrolysates of porcine blood plasma protein. J. Korean Soc. Appl. Biol. Chem..

[38] Ghisaidoobe AB, Chung SJ (2014). Intrinsic tryptophan fluorescence in the detection and analysis of proteins: a focus on Förster resonance energy transfer techniques. Int. J. Mol. Sci..

[39] Zhou J, Wang X, Ai T, Cheng X, Guo HY, Teng GX, Mao XY (2012). Preparation and characterization of β-lactoglobulin hydrolysate-iron complexes. J. Dairy Sci..

[40] Knappskog PM, Haavik J (1995). Tryptophan fluorescence of human phenylalanine hydroxylase produced in *E. coli*. Biochemistry.

[41] Miranda FF, Kolberg M, Andersson KK, Geraldes CF, Martínez A (2005). The active site residue tyrosine 325 influences iron binding and coupling efficiency in human phenylalanine hydroxylase. J. Inorg. Biochem..

[42] Uppal R, Lakshmi KV, Valentine AM (2008). Isolation and characterization of the iron-binding properties of a primitive monolobal transferrin from *Ciona intestinalis*. J. Biol. Inorg. Chem..

[43] Wu HH, Liu ZY, Zhao YH, Zeng MY (2012). Enzymatic preparation and characterization of iron-chelating peptides from anchovy (*E. japonicus*) muscle protein. Food Res. Int..

[44] Van Der Ven C, Muresan S, Gruppen H, De Bont DB, Merck KB, Voragen AG (2002). FTIR spectra of whey and casein hydrolysates in relation to their functional properties. J. Agric. Food Chem..

[45] Lin J, Cai X, Tang M, Wang S (2015). Preparation and evaluation of the chelating nanocomposite fabricated with marine algae *Schizochytrium* sp. protein hydrolysate and calcium. J. Agric. Food Chem..

[46] Nara M, Morii H, Tanokura M (2013). Coordination to divalent cations by calcium-binding proteins studied by FTIR spectroscopy. Biochim. Biophys. Acta..

[47] Zhao L, Huang S, Cai X, Hong J, Wang S (2014). A specific peptide with calcium chelating capacity isolated from whey protein hydrolysate. J. Funct. Foods..

[48] Chen D, Liu Z, Huang W, Zhao Y, Dong S, Zeng M (2013). Purification and characterisation of a zinc-binding peptide from oyster protein hydrolysate. J. Funct. Foods..

[49] Bhat ZF, Kumar S, Bhat HF (2015). Bioactive peptides of animal origin: a review. J. Food Sci. Technol..

[50] Lebetwa N, Suzuki Y, Tanaka S, Nakamura S, Katayama S (2019). Enhanced anti-allergic activity of milk casein phosphopeptide by additional phosphorylation in ovalbumin-sensitized mice. Molecules.

[51] Reynolds EC, Riley PF, Adamson NJ (1994). A selective precipitation purification procedure for multiple phosphoseryl-containing peptides and methods for their identification. Anal. Biochem..

[52] Huang SL, Zhao LN, Cai X, Wang SY, Huang YF, Hong J, Rao PF (2015). Purification and characterisation of a glutamic acid-containing peptide with calcium-binding capacity from whey protein hydrolysate. J. Dairy Res..

[53] Choi DW, Lee JH, Chun HH, Song KB (2012). Isolation of a calcium-binding peptide from bovine serum protein hydrolysates. Food Sci. Biotechnol..

[54] Lv Y, Bao X, Liu H, Ren J, Guo S (2013). Purification and characterization of calcium-binding soybean protein hydrolysates by Ca^2+^/Fe^3+^ immobilized metal affinity chromatography (IMAC). Food Chem..

[55] Schmidt S, Reinecke A, Wojcik F, Pussak D, Hartmann L, Harrington MJ (2014). Metal-mediated molecular self-healing in histidine-rich mussel peptides. Biomacromol.

[56] Fan, W., Wang, Z., Mu, Z., Du, M., Jiang, L., EI-Seedi, H. R., & Wang, C. Characterizations of a food decapeptide chelating with Zn (II). *eFood*. **1**(4), 326–331. DOI:10.2991/efood.k.200727.001 (2020).

[57] Hou H, Wang S, Zhu X, Li Q, Fan Y, Cheng D, Li B (2018). A novel calcium-binding peptide from Antarctic krill protein hydrolysates and identification of binding sites of calcium-peptide complex. Food Chem..

[58] Sambuy Y, De Angelis I, Ranaldi G, Scarino ML, Stammati A, Zucco F (2005). The Caco-2 cell line as a model of the intestinal barrier: influence of cell and culture-related factors on Caco-2 cell functional characteristics. Cell Biol. Toxicol..

[59] Lin YL, Cai XX, Wu XP, Lin SN, Wang SY (2020). Fabrication of snapper fish scales protein hydrolysatecalcium complex and the promotion in calcium cellular uptake. J. Funct. Foods..

[60] Hou T, Liu W, Shi W, Ma Z, He H (2017). Desalted duck egg white peptides promote calcium uptake by counteracting the adverse effects of phytic acid. Food Chem..

[61] Hou T, Wang C, Ma Z, Shi W, Weiwei L, He H (2015). Desalted duck egg white peptides: promotion of calcium uptake and structure characterization. J. Agric. Food Chem..

[62] Daengprok W, Garnjanagoonchorn W, Naivikul O, Pornsinlpatip P, Issigonis K, Mine Y (2003). Chicken eggshell matrix proteins enhance calcium transport in the human intestinal epithelial cells, Caco-2. J. Agric. Food Chem..

[63] Liu H, Lv Y, Xu J, Guo S (2017). Interaction mode of calcium-binding peptides and Caco-2 cell membrane. Food Res. Int..

[64] Sakdasri, W., Ngamprasertsith, S., Sooksai, S., Noitang, S., Sukaead, W., & Sawangkeaw, R. Defatted fiber produced from lemon basil (*Ocimum citriodorum* Vis.) seed with supercritical CO_2_: Economic analysis. *Ind. Crops Prod.* **135**, 188–195. DOI:10.1016/j.indcrop.2019.03.042 (2019).

[65] Bradford MM (1976). A rapid and sensitive method for the quantitation of microgram quantities of protein utilizing the principle of protein-dye binding. Anal. Biochem..

